# The Epidemiological and Histopathological Profiling of Basal Cell Carcinoma: Insights from a 4-Year Institutional Cohort in a Romanian Clinical County Hospital

**DOI:** 10.3390/diagnostics15182377

**Published:** 2025-09-18

**Authors:** Iuliu-Gabriel Cocuz, Martin Manole, Maria-Cătălina Popelea, Raluca Niculescu, Maria Elena Cocuz, Adrian Horațiu Sabău, Andreea Cătălina Tinca, Andreea Raluca Cozac-Szőke, Diana Maria Chiorean, Alexandru Constantin Ioniță, Eugenia Corina Budin, Georgian-Nicolae Radu, Emoke Andrea Szasz, Ovidiu Simion Cotoi

**Affiliations:** 1 Pathophysiology Department, “George Emil Palade” University of Medicine, Pharmacy, Sciences and Technology of Targu Mures, 540142 Targu Mures, Romania; iuliu.cocuz@umfst.ro (I.-G.C.); raluca.niculescu@umfst.ro (R.N.); adrian-horatiu.sabau@umfst.ro (A.H.S.); andreea-catalina.tinca@umfst.ro (A.C.T.); andreea-raluca.szoke@umfst.ro (A.R.C.-S.); chioreandianamaria@yahoo.com (D.M.C.); corina.budin@umfst.ro (E.C.B.); ovidiu.cotoi@umfst.ro (O.S.C.); 2 Clinical Pathology Department, Mures Clinical County Hospital, 540011 Targu Mures, Romania; popelea.maria@gmail.com (M.-C.P.); george.radu098@gmail.com (G.-N.R.); emoke.szasz@umfst.ro (E.A.S.); 3 Faculty of Medicine, “George Emil Palade” University of Medicine, Pharmacy, Sciences and Technology of Targu Mures, 540142 Targu Mures, Romania; alexionita2@gmail.com; 4 Histology Department, “George Emil Palade” University of Medicine, Pharmacy, Sciences and Technology of Targu Mures, 540142 Targu Mures, Romania; 5 Fundamental Prophylactic and Clinical Disciplines Department, Faculty of Medicine, Transilvania University of Brasov, 500003 Brasov, Romania; maria.cocuz@unitbv.ro; 6 Clinical Pneumology and Infectious Diseases Hospital of Brasov, 500174 Brasov, Romania; 7 Pneumology Department, Mures Clinical County Hospital, 540011 Targu Mures, Romania

**Keywords:** basal cell carcinoma, histopathology, epidemiology, topographical distribution

## Abstract

**Background/Objectives**: Basal cell carcinoma (BCC) of the skin is a type of non-melanocytic skin cancer. The European incidence of non-melanocytic skin cancers is 14.2 per 100,000 people, with a mortality rate of 0.5, thus ranking Europe third in the world in terms of incidence and mortality rate, according to the WHO Global Cancer Observatory. The objective of this study was to highlight the histological, epidemiological, and clinicopathological aspects of BCCs diagnosed in the Clinical Pathology Department of the Mures Clinical County Hospital between January 2021 and December 2024. **Methods**: We performed a retrospective, descriptive, observational study between January 2021 and December 2024 in the Mureș Clinical County Hospital, Targu Mureș, Romania, by analysing data from histopathological reports and histological slides from patients with a positive diagnosis of BCC. The inclusion criteria for this study consisted of patients who presented a histopathological diagnosis of BCCs during the study period. Lesions were divided into two study cohorts—a general cohort and head and neck cohort. The collected data included epidemiological data, macroscopic features, and microscopical characteristics. **Results**: A total of 540 lesions were included in this study (general cohort), of which 395 were included in the head and neck cohort. This study revealed a higher incidence of BCC in 2024, affecting mostly urban patients (*p* < 0.001), with more aggressive forms (*p* < 0.001). The tumours found among males (*p* = 0.0189) and in rural patients (*p* = 0.0126) were bigger, but the tumoural volumes decreased over time (*p* < 0.001). The mixed form of BCC was associated with more aggressive histological subtypes (*p* < 0.001). **Conclusions**: BCC presents variability depending on age, gender, environment of origin, and topography, as well as histological subtype and aggressiveness, thus highlighting the need for a personalised approach in terms of diagnostics and treatment.

## 1. Introduction

Basal cell carcinoma (BCC) is a type of non-melanocytic skin cancer (NMSC). BCC is a malignancy of the skin originating in the basal layer of the epidermis. It develops from pluripotent stem cells in the hair follicles or interfollicular dermis without producing precancerous lesions. BCC tends to occur in areas exposed to UV radiation over a long period of time, with a predilection for the head and neck, followed by the thoracic region and the upper and lower limbs. In the literature, rare cases of BCC occurring on mucosa and soles have also been reported [1].

The European incidence of non-melanocytic skin cancers is 14.2 per 100,000 people, with a mortality rate of 0.5, thus ranking Europe third in the world in terms of incidence and mortality rate according to the WHO Global Cancer Observatory [2]. In Romania, the incidence is 8.6 per 100,000 inhabitants, according to the WHO Global Cancer Observatory [2]. A closer analysis of these data shows that men have a much higher incidence and mortality rate than women. While the incidence in women is only 5.8, that in men is more than double, at 12.3, and the mortality rate for women is only 0.66 compared with 1.7 for men, which is almost 3 times greater [2].

Over 26 different subtypes of BCC are mentioned in the literature [3], each with distinct clinical and histological features [4,5]. Of these, the most common and frequently diagnosed histopathological subtypes are the nodular, micronodular, superficial, pigmented, infiltrative, and fibroepithelial (Pinkus fibroepithelioma), infundibulocystic, sclerosing (morpheaform-like), basosquamous or metatypical, and sarcomatoid subtypes [6]. Ulceration may be present within these lesions. A lesion may also contain several of the subtypes listed above [1,5,6].

In addition to the standard classification of BCC, an important characteristic of this type of skin lesion is the absence of melanin. However, some subtypes may have minimal traces of melanin [5].

**Figure 1:**
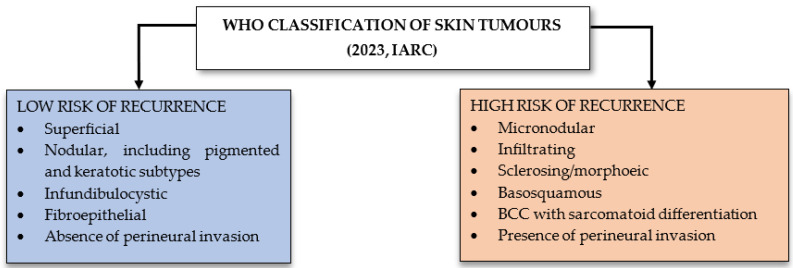
Risk of recurrence according to WHO [3] classification.

The WHO classifies (Figure 1) BCC according to the risk of tumour recurrence [3]. Thus, the superficial, nodular, pigmented, infundibulocystic, and fibroepithelial subtypes are included in the low risk of tumour recurrence category, while the subtypes sclerosing/morpheaform, infiltrative, basosquamous, sarcomatoid, and micronodular are included in the high risk of recurrence category. According to the 2023 classification, the presence or absence of perineural invasion may represent a risk factor. TNM staging is not used in the diagnosis of BCC due to the extremely low risk of metastasis [1,6].

The pathophysiology of BCC is complex and is determined by the interaction between environmental and individual factors. Among the most important aetiologies of BCC are prolonged exposure to UV rays, sunburns at a young age, and mutations in the embryonic phase of the Hedgehog (HH) pathway, which result in malignant tissue proliferation in BCC.

**Figure 2:**
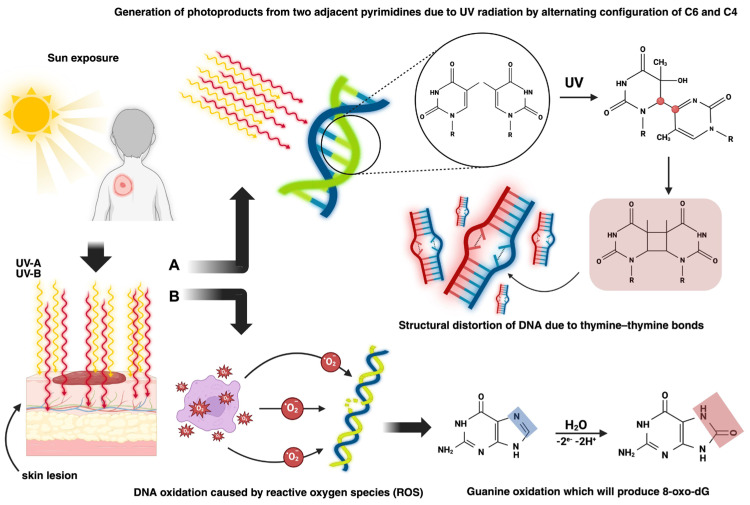
Indirect DNA lesion caused by UVA and UVB rays (reactive oxygen species (ROS) oxidise guanine to 8-oxo-deoxyguanosine) and direct DNA lesion caused by the formation of photoproducts that alter the configuration of carbon 6 and 4, which distort thymine–thymine bonds [11]. A—Direct damage. B—Indirect damage. Created in BioRender. Manole, M. (2025) https://BioRender.com/ [11].

In terms of pathophysiologic mechanisms, there are two pathways through which BCC can develop (Figure 2): exogenous causes such as UV radiation, which leads to both direct skin lesions due to the formation of cyclobutene pyrimidine dimers (CPDs) and photoproducts and indirect skin lesions produced by the interaction of reactive oxygen species (ROS) with DNA, and endogenous causes, primarily genetic mutations [7,8,9,10].

**Figure 3:**
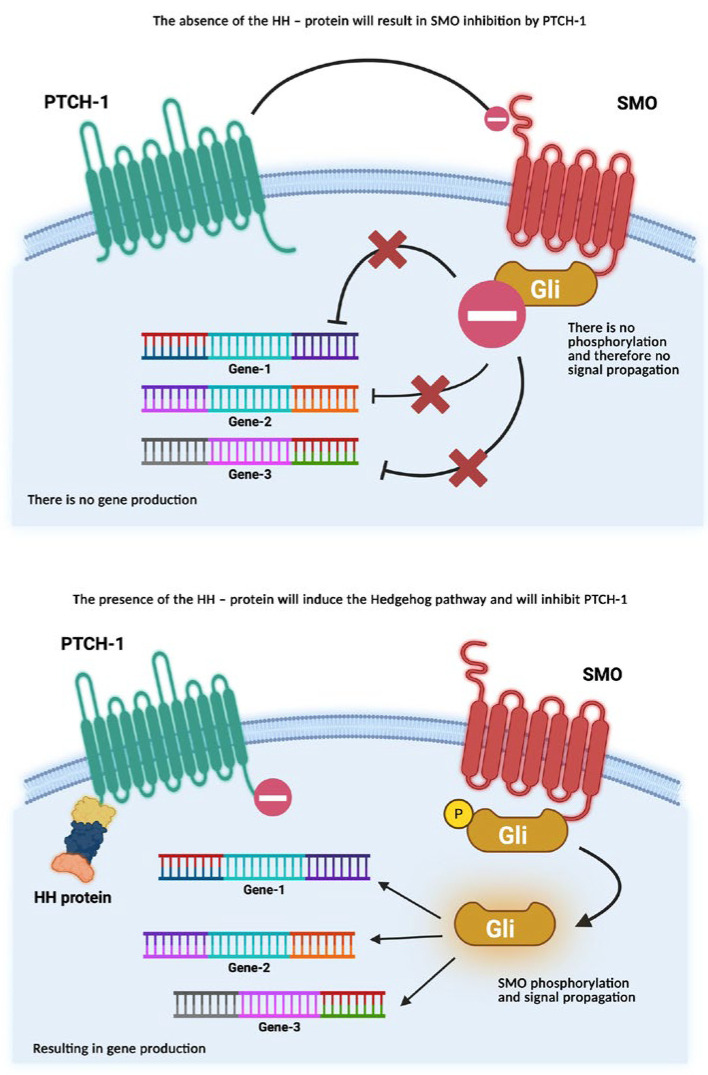
Activated and inhibited Hedgehog pathway (PTCH1 = patch receptor 1; SMO = smoothen receptor; P = phosphorus; Gli = transcription factors). Original figure—Martin Manole [8,9,15]. Created in BioRender. Manole, M. (2025) https://BioRender.com/ [16].

According to the concentrations of the Hedgehog protein (P-HH), in the Hedgehog (HH) pathway (Figure 3), the synthesis of different genes is induced. Even if the HH pathway plays an important role in embryonic development, it can also influence tumoural homeostasis [12,13,14,15].

In the Canonical Hedgehog pathway (CHHP), the action of three types of P-HH occurs: Sonic (SHH), Indian (IHH), and Desert (DHH). The expression of these ligands is tissue-dependent. When one of the ligands binds to PTCH, SMO will no longer be inhibited and can phosphorylate and induce the transcription of Gli factors (Gli-1-activator, Gli-2 and Gli-3-suppressor), which is related to cell development and differentiation [8,9,15].

The non-canonical Hedgehog pathway (nCHHP) can be SMO-independent, in which case both PTCH1-PHH-mediated and SMO-dependent cell proliferation affect calcium channels, chemotacticity, and cell migration, thus leading to tumoural survival and proliferation [8,9,15].

BCC treatment requires a multidisciplinary and individualised approach. The treatment management strategy involves considering a variety of factors, such as location, size, and histological subtype, which may indicate a possible outcome.

Even though surgical excision is the gold standard, topical treatments with Imiquimod or 5-Fluorouracil may be considered depending on the situation when the BCC subtype has a low risk of recurrence and is less than 2 mm deep. In the case of inoperable lesions, radiotherapy and treatment with local destructive procedures such as cryotherapy or laser therapy may be taken into consideration.

Systemic treatment consists of targeted therapies against the Hedgehog pathway, represented by Hedgehog pathway inhibitors (iHHs). This treatment is used primarily in advanced BCC and when radiotherapy and surgery are not feasible options. Two molecules are approved for this category: Vismodegib, which inhibits the SMO pathway, and Sonidegib, which is similar to Vismodegib [17].

The particularity of this study is emphasised by the high number of cases included, after the COVID-19 pandemic. Moreover, all the parameters that were analysed represent a unique combination of epidemiological and demographic characteristics in correlation with macroscopic appearance and histological characteristics. Even though in comparison to other malignancies, BCC is not as life-threatening as other cancers, this study presents correlations that might reveal a predictive pathway of evolution for each patient.

The objective of this study was to highlight the histological, epidemiological, and clinicopathological aspects of BCCs diagnosed in the Clinical Pathology Department of the Mures Clinical County Hospital between January 2021 and December 2024.

## 2. Materials and Methods

This was a retrospective, descriptive, and observational study conducted within the Clinical Pathology Department of the Mureș Clinical County Hospital, Targu Mureș, Romania, by analysing data from reports and histopathological slides from patients who presented a positive diagnosis of BCC.

A total of 540 lesions originating from different surgical and non-surgical wards were included in this study, which showed different macroscopic shapes of both excised fragments and tumours.

The inclusion criteria for this study consisted of patients who presented a histopathological diagnosis of BCC during the designated study period (January 2021–December 2024). The exclusion criteria consisted of all skin lesion diagnoses that were not BCC and all patients who presented a positive diagnosis of basal cell carcinoma outside the study period. This study was conducted in accordance with the Declaration of Helsinki and approved by the Institutional Ethics Committee of Mures Clinical County Hospital (18310/23 January 2025).

Microsoft Excel iOS v.16 was used for data management. The data collected were as follows: epidemiological data (year of diagnosis, patient’s age and gender, patient’s environment of origin—rural or urban), macroscopic features (excision site, lesion’s shape and size), microscopical characteristics (histological subtype, the presence or absence of recurrence, the presence or absence of different types of differentiation or the presence or absence of ulceration, the presence or absence of peritumoural inflammatory infiltrate, and the presence or absence of perivascular invasion or excision of the tumour formation within safe surgical limits). BCCs were grouped into simple (single histologic subtype) and mixed (more than one histologic subtype) groups.

The lesion size was converted to tumour (or excision) volume using the following calculation formula: length × width × height. When the value of height and/or width/length was missing, a value of 1 mm was arbitrarily assigned. Volumes that did not have sufficient data or at least 2 out of the 3 necessary dimensions for the calculation were excluded.

Considering the large number of head and neck formations in this study, the tumours were divided into two cohorts: the general cohort, consisting of all BCCs included in this study, and the head and neck cohort, including only BCCs in this area. The areas of tumour excision were grouped according to anatomical regions as follows: the head and neck region, anterior and posterior thoracic region, abdomino-lumbar region, upper and lower limb regions, inguinal region, perineal region, and pelvic region. Histopathological reports that did not show an excision area were put into the Unspecified category. Head and neck excision sites were further grouped as follows: Cervical (neck regions), facial (excluding the frontal region), nasal, ocular, auricular, and scalp (including the frontal region).

Statistical data analysis was performed using IBM SPSS Statistics software, version 30. The data distribution was checked by evaluating Kurtosis and Skewness parameters and considering histograms and Q-Q plots, and it corroborated Shapiro–Wilk’s test for distribution. Thus, these all confirmed the non-parametric distribution of the data. The quantitative data assessed were expressed as the median, together with the minimum–maximum range (median (min-max)), and the qualitative data were expressed as absolute values, together with relative values (n(%)).

To analyse statistically significant differences, the Spearman test for correlations of non-parametric continuous variables and the Mann–Whitney U and Kruskal–Wallis tests for comparisons of non-parametric continuous variables were performed. For categorical data, Chi^2^, Monte Carlo, or Fisher’s test was used where appropriate. When more than one statistical test was performed for the same data set, the Bonferroni correction (*p′*) was applied to avoid type I false positive errors, according to the following formula:

$$p^{\prime }\;=\;p\;(\mathrm{significance}\mathrm{number})\;\times \;m\;(\mathrm{number}\mathrm{of}\mathrm{tests}\mathrm{performed})$$

The odds ratio (OR) was calculated where appropriate with the 95% confidence interval used in medical studies (CI = 95%). The statistical significance threshold was set at α = 0.05. Only values that had relevant statistical significance were selected. For the calculation of statistical differences, an equal distribution was arbitrarily considered to obtain theoretical *p*-values that were deemed necessary.

To perform a statistical analysis of the categories with low frequencies (macroscopic shape, histological features, etc.), data were grouped into the category “others”.

Excel, IBM SPSS v.30, GraphPad Prism 10, and Python v.3.10 were used to perform statistical tests. Figures in the Introduction Section were generated using BioRender to visually evidence and enhance the comprehension of the underlying pathophysiological processes.

## 3. Results

Table 1 shows the numerical and percentage distribution of both simple and mixed BCC lesions in terms of histological subtype and tumour characteristics, for the general cohort and head and neck cohort. (NA—not assessed, due to low-frequency categories).

**Figure 4:**
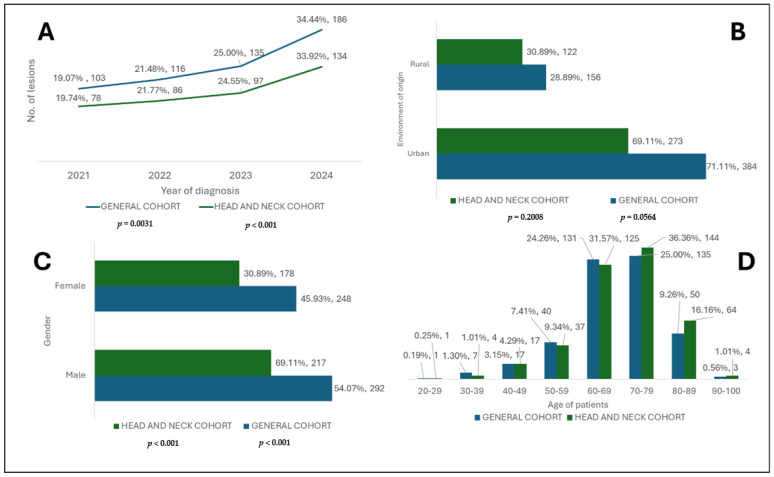
Numerical and percentage distribution of year of diagnosis and epidemiological data of patients with BCC included in the study (general cohort and head and neck cohort). (**A**) Year of diagnosis. (**B**) Environment of origin. (**C**) Gender. (**D**) Age.

Figure 4 illustrates the numerical and percentage distribution of the year of diagnosis and epidemiological data of patients with BCC included in this study (general cohort and head and neck cohort).

**Figure 5:**
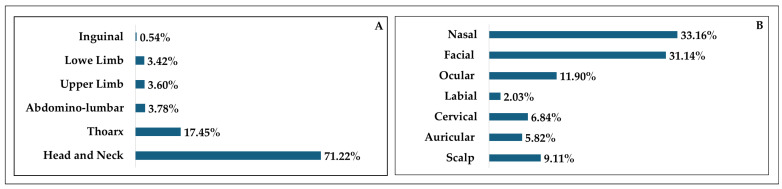
Percentage distribution of topographical sites for BCC. (**A**) Percentage distribution of basal cell carcinoma cases in the general cohort. (**B**) Percentage distribution of basal cell carcinoma cases in the head and neck cohort.

Figure 5 illustrates the percentage distribution of BCC cases according to the anatomical location.

Table 2 presents the distribution and statistical analysis of the histological subtype in relation to the simple or mixed type of BCC in the general cohort and head and neck cohort.

Table 3 shows the distribution and analysis of the histological subtype in terms of the patients’ gender and their environment of origin, in both the general cohort and the head and neck cohort. This table further analyses the distribution of all subtypes according to the excisional region in the general cohort under three major categories (head and neck, thorax, and other regions), with only significant *p*-values being presented, as well as those close to significance. A full table of all values can be found in the Supplementary Materials.

Table 4 shows an analysis of the excision sites of the general cohort with the following variables: age, gender, environment of origin, year of diagnosis, histological subtype, histological characteristics, and macroscopic shapes of excisions and tumours.

Table 5 analyses the excision areas in the head and neck cohort in relation to the following: histological subtypes, tumoural characteristics, and the statistical analysis of the excisional shapes in relation to the excision site and histological characteristics of the tumours. For further details and a full table of all values, please refer to the Supplementary Materials of this paper.

The top of Table 6 presents the numerical and percentage distribution of the tumoural and excisional volumes in both cohorts. Furthermore, the distribution of tumoural and excisional volumes by gender (Male/Female), environment of origin (Rural/Urban), and year of diagnosis (from 2021 to 2024) in the general cohort and head and neck cohort can be found in the Supplementary Materials. At the bottom of this table, the correlations of tumoural and excision volumes in relation to the patients’ age and the year of diagnosis are shown for both the general cohort and head and neck cohort.

Table 7 shows a multiple volumetric analysis in both the general and head and neck cohorts. The simple and mixed types of BCC were examined in relation to both tumoural and excisional volumes, and excisional sites were also considered with respect to each specific area.

Table 8 analyses the age distribution in the head and neck cohort in relation to excisional sites. This table reveals the highest and lowest median ages. For further details and a full table of all values, please refer to the Supplementary Materials of this paper.

Table 9 shows the tumoural and excisional volumes of both the general and head and neck cohorts according to the histological subtype and features of BCC. For further details and a full table of all values, please refer to the Supplementary Materials of this paper.

## 4. Discussion

BCC is a non-melanocytic skin cancer (NMSC), and it represents one of the most frequently diagnosed malignant skin lesions. Due to its paradoxical characteristics regarding the behaviour of a malignant tumour, it rarely results in metastases, and it has a mortality rate close to zero; therefore, its excision represents a type of curative therapy [18,19,20].

As a result, the study of the epidemiological and histopathological aspects and the treatment of this malignancy does not garner the same scientific interest as in the case of other malignant lesions with much higher lethality, such as glioblastoma and small-cell lung carcinoma [18,19,20,21].

The highest incidence of BCC was registered in 2024 in both cohorts, representing more than a third of all cases (Figure 4). This fact reflects the early screening of these lesions and a rising incidence. It can also be affirmed that the tendency of cases to be extended along with the end of the emergency state related to the COVID-19 pandemic suggests the return to normality of the healthcare system [22,23,24,25,26]. Furthermore, climate change might also play a crucial role. Year after year, Romania registers a constant increase in temperature and critical UV sun exposure conditions and even death according to the National Institute of Meteorology (INM). If we also consider that Romanian citizens are free to move to sunnier places, this would ultimately contribute to the risks and DNA damage described [27].

The environment of origin of the patients was significantly represented as urban areas (*p* < 0.001) in both analysed cohorts. This aspect can be explained by the fact that patients from rural areas have less access to specialised medical services, and often, points of care are located at considerable distances from them compared to those from urban areas who generally benefit from easier access [26,28,29].

The gender distribution of BCC between male and female patients was not significant, but a slight male predominance was observed (Figure 4), which is consistent with the specialised literature [30]. Furthermore, according to another study, even if at risk, males showed a positive perception about tanning, and men are mostly diagnosed with NMSC and melanocytic skin cancers [31].

The analysis of age groups revealed a higher incidence in elderly patients, especially those in their 8th decade of life (70–79 years) in the general cohort and 7th decade (60–69 years) in the subgroup. These data underline the hypothesis of BCC carcinogenesis, in which the accumulation of cellular DNA damage caused by UV rays leads to damage, especially in elderly patients who have a substantially reduced DNA repair capacity [32,33]. As shown in Figure 4, regarding the categories of younger patients, there is a relatively lower number of lesions, and the incidence starts to increase in patients over 40 years of age.

The analysis of BCC types revealed a significant predominance of the mixed form (Table 1) in both groups (*p* < 0.001), which reconfirms the morphological and architectural complexity of BCC [34,35,36]. The simple or mixed form was not influenced by the patient’s gender, thus suggesting that gender cannot be considered a risk factor when it comes to a specific type of BCC, although a higher but insignificant incidence (*p* > 0.05) of mixed BCC was observed among men [34,37,38,39].

The nodular histological subtype was the most common among the simple and mixed forms of BCC in both cohorts (Table 1). The main difference was seen while analysing the second and third most common subtypes. The superficial and infiltrative types were observed in simple BCC, while the adenoid cystic and superficial ones were mainly observed in mixed BCC, and this remained the same in both cohorts. A comparative analysis of the simple and mixed types of lesion characteristics (Table 1) showed an increased rate of tumour-free resection margins in both the general and head and neck cohorts, which is beneficial for patients.

While analysing these simple and mixed forms regarding lesion characteristics, in both the general and head and neck cohorts, the samples presented a high percentage of free resection margins, with slightly better results in the head and neck (Table 1). With the complete excision of the lesion and free resection margins, patients no longer need to undergo further surgery, as in nearly all cases, this represents a curative intent [7,26,40].

From a microscopic point of view (Table 1), the peritumoural inflammatory infiltrate was largely present in both forms (simple and mixed BCC), as well as in the cohorts. A difference between BCC types was suggested by the presence of differentiations, squamous, sebaceous, and pillar, which were almost entirely present in the mixed forms. Ulcerations were present in a considerably higher percentage in the mixed rather than simple BCC form, which was true for both study groups [26]. The recurrence of BCC was rare for both types—simple and mixed—independently of the analysed cohort, which is a rarity that is also confirmed by the literature [41,42].

In terms of excisional sites in the general cohort (Figure 5), it is largely seen that the head and neck area was mainly affected, followed by the thoracic region. The least affected area was the inguinal one as it is inevitably one of the most covered areas of the human body, thus shielding it from sun damage. More in depth, the head and neck cohort (Figure 5) presented a heterogeneous distribution of excisional sites. The nasal area was mainly affected, followed by the facial regions. Interestingly, the labial area saw the lowest incidence, which might be a result of the predominance of squamous cell carcinomas rather than BCC. Furthermore, lip balms and other beauty products like lipsticks might offer slight protection against sunburns [41].

The occurrence of histological subtypes and tumour characteristics in the simple and mixed forms of BCC were analysed in both groups (Table 2), revealing statistically significant associations.

It was shown that the nodular subtype is significantly more common in the mixed form, which is consistent with other studies [43]. The main difference was seen from a cohort point of view. The chance of a nodular subtype being found in mixed BCC in the head and neck was greater than 3 times more likely (OR—3.14 *p* < 0.001), while in the general cohort, it was double this, at 6 times more likely (OR—6.53 *p* < 0.001).

The micronodular subtype was present almost exclusively in the mixed form (*p* < 0.001) when analysing the general cohort. It was only found in the mixed form in the head and neck, and therefore, it presented an odds ratio tending towards infinity [44,45].

The infiltrative subtype was observed in both the general (OR—3.58/*p* = 0.005) and head and neck subgroups (OR—4.36/*p* = 0.01), in which the rates of occurrence were 3 and 4, respectively. Similar results were obtained in two different studies, thus suggesting that the mixed type is associated with more aggressive forms [34,38].

A significant association was noted (*p* < 0.001) between the mixed form of BCC and pillar differentiation, with a chance of occurrence over 5 times as high within this form in both cohorts. All these findings thus point to the architectural complexity and increased aggressiveness of the mixed type of BCC, thereby corresponding with previous research [45].

The distribution of histological subtypes according to gender (Table 3) did not show significant differences (*p* > 0.05) in any of the analysed groups, even though some subtypes, such as the infundibulocystic and pigmented ones, were predominantly present in men, and others, such as the Pinkus subtype, were found exclusively in women. Additionally, this occurred only in the thoracic region (Table 3), but it was above the significance threshold. Table 3 also presents the incidence of the superficial histological subtype, in which the most affected area was the thoracic region [39,46,47].

The analysis of anatomical and clinical parameters in relation to the excision sites (Table 4) of the general cohort revealed a predominant involvement of the head and neck area but no significant differences in terms of patient age or gender (*p* > 0.05) [48]. However, there were significant differences within the general cohort in terms of environment of origin (*p* = 0.0037). Even if those from both environments were predominantly affected in the head and neck area, urban patients had higher rate of chest-involved lesions compared to those from rural areas [19,48]. Furthermore, the superficial histological subtype had a significantly higher rate in the thoracic region compared to the other regions (*p* < 0.001; the category “other regions” had the highest rate, but the number of cases in every distinct area is smaller, so we considered the thoracic region to be predominant), as shown in Table 3 and Table 4.

The histological characteristics and subtypes vary significantly depending on where the lesion is localised within the head and neck cohort (*p* < 0.001), as shown in Table 5. Although the nodular subtype was found in all excision sites, it predominantly affected the nasal and facial areas, while the superficial subtype showed a predilection for the same areas [49]. The infiltrative subtype was mainly observed in the nasal and facial areas, which can be explained by the fact that these regions of the head and neck are the most exposed to UV radiation, regardless of the time of day [50].

Histological characteristics such as non-infiltrated tumour margins and ulcerations also varied significantly (Table 5), depending on the excision area. Ulcerations were predominantly seen in the ocular region, followed by the auricular region. This may reflect the difficulty encountered in certain anatomic areas during surgery such as the ear, in which the lowest percentage of free margins was observed [51,52].

The shape of the excision within the head and neck (Table 5), as in the case of the general cohort, showed a significant association between elliptical excisional shape and free margins (*p* < 0.001), thus indicating better surgical control [53,54]. The relationship between the macroscopic shape of the tumours and the excision areas (Table 5) showed significant associations, as suggested by the fact that the nodular subtype was mainly associated with nodular and elevated shapes [55], while the adenoid cystic and superficial subtypes were often associated with irregular shapes.

Ulcerated lesions were frequently observed in the nodular subtype [6], followed by the adenoid cystic and infiltrative subtypes, which again suggests the aggressiveness and different behaviours of certain subtypes [56].

The volumetric analysis of BCC (Table 6) showed that in the general cohort, the tissue samples predominantly had volumes between 200 and 499 mm^3^, and the tumours found in these samples predominantly had volumes smaller than 50 mm^3^. These findings were also observed in the head and neck cohort even though there were slightly fewer lesions between 500 and 999 mm^3^ and over 1000 mm^3^, thus indicating that locations outside this subgroup have larger volumes. At the same time, the insidious nature of their appearance, marked by slow progression and reduced visibility in certain areas like the thorax or the limbs, leads to a delay in patients seeking medical attention and therefore the need for a larger excision to obtain tumour-free margins [49,57,58].

Following the analysis of tumour and excision volumes according to gender, environment of origin, and year of diagnosis in both cohorts (Table 6), it was observed that the median volumes showed significant differences in relation to certain parameters.

The gender of the patients did not influence the median tumoural volume, neither in the general cohort (*p* = 0.4158) nor in the subgroup (*p* = 0.6327), but men had significantly higher median volumes of excised pieces both in the general cohort (*p* = 0.0189) and in the subgroup (*p* = 0.0077) [59], which may suggest a tendency for them to develop lesions with larger affected areas (Table 6).

The median tumoural volumes of the head and neck subgroup (Table 6) were significantly higher (*p* = 0.0126) among rural patients. The general cohort also presented higher median tumoural volumes for rural patients, but the difference was only close to significance (*p* = 0.0764). A further analysis of the difference in terms of the median volume of excised tissue samples in both cohorts revealed an insignificant *p*-value for both the general (*p* = 0.8875) and head and neck (*p* = 0.1907) cohorts. Rural patients tend to have higher tumoural volumes and excisions, and it has also been widely reported that rural patients are diagnosed later, which can ultimately lead to higher tumoural volumes and dimensions compared to urban patients who have faster and easier access to healthcare providers, which might also be the case in Romania [26,31].

An analysis of the year of diagnosis suggested a significant downward trend in median tumour volumes in both the general cohort (*p* < 0.001, decrease of 69.7%) and the subgroup (*p* < 0.001, decrease of 62.7%), as can be seen in Table 6. Even though the same was found in the case of excised tissue volumes, the differences were insignificant. This decreasing trend over the years most likely indicates a better screening campaign or easier access to treatment for skin lesions; in a digitalised world, access to information is easier and awareness can be easily raised [60].

Table 6 supports and reinforces the observations identified. A significant negative correlation was observed in terms of tumoural volumes and the year of diagnosis, in both the general cohort and subgroup, which supports the hypothesis of earlier screening and the improved quality of medical services in this direction [61]. The same significant negative correlation is also repeated in the case of excised tissue volumes in both groups. In terms of age, both the general and head and neck cohorts presented a positive correlation with tumour and excised tissue sample volumes. This indicates that older patients tend to have larger excisional volumes and therefore require more extensive resections and care, as supported by the literature [62].

Tumour and excision volumes were analysed in relation to the forms of BCC (Table 7) in both cohorts. Mixed BCC presented higher median volumes in terms of both tumour and excised tissue samples, in both the general and head and neck groups. This shows that the type of BCC (simple or mixed) does not influence tumour or excision volume. The literature suggests that the mixed type tends to be significantly larger than other forms of BCC [34,63], and these differences may be the result of different patient samples or even larger groups of patients. In our study, the differences found were insignificant; only the tumour volumes in the general cohort had a *p*-value close to significance (*p* = 0.0889).

In the general cohort, both tumoural and excised tissue sample volumes were analysed in relation to the excisional sites. Significant differences were found in both cases (*p* = 0.0043 and *p* < 0.0001) (Table 7), and the largest medians were found in the thoracic region compared to other regions. This reaffirms that less obvious lesions tend to be identified later and therefore lead to larger volumes at the time of diagnosis [64,65]. Continuing the same volumetric analysis in the head and neck cohort, significant differences were identified in terms of excisional sites and the volumes of both tumours (*p* = 0.0681) and excised tissue samples (*p* < 0.0001) (Table 7).

Labial-located lesions had the highest median volume in both tumoural and excision samples. These aspects are found in the medical literature, which confirms the rarity of labial cases, but larger volumes are present at this level [66]. Nasal BCC presented the smallest median volume of excised tissue samples, while auricular region lesions presented the smallest median tumoural volume [67]. The age of patients showed significant differences (*p* = 0.0172) in terms of the excision area in the head and neck cohort (Table 8). The highest median age was found in labial excisions, while the lowest was observed in the facial region. This leads to the fact that older people tend to not only have more frequent labial lesions but also present larger tumoural volumes that often result in larger excisions as well.

The tumour volume and the number of pieces in the groups were analysed in relation to histological subtypes and tumour characteristics (Table 9). In the general cohort, no statistically significant values were found with the subtypes or characteristics, in terms of either tumour or excisional samples (*p* > 0.05). Regarding the head and neck subgroup, the *p*-values of the excisional volumes with both parameters were above 0.05. The same was observed for tumour volumes, but their analysis with histological subtypes was close to statistical significance (*p* = 0.0573). The highest median in the general cohort was observed in BCC that presented trichilemmal differentiation, while the smallest median was observed in pigmented BCC [68,69].

Study limitations: Our study has several limitations. It is a retrospective study, and the variables of interest or confusion factors cannot be controlled. Furthermore, this study was conducted in a single centre on BCC cases at the Mureș County Clinical Hospital, which may lead to geographical bias. Furthermore, certain data, such as lesion volumes, were calculated arbitrarily when only two dimensions were available, which may introduce a measurement error. For lesion types that had low frequencies, they were grouped into the “others” category, which may lead to a loss of granularity. Additionally, for certain parameters, such as certain histological subtypes or anatomical areas, there were sparse data in both cohorts. Thus, there may be a significant reduction in statistical power. Another limitation is that there is no data on exposure to risk factors or the follow-up of these patients.

## 5. Conclusions

Our study revealed a more pronounced increase in BCC in the head and neck cohort than in the general cohort, with an age difference between the two cohorts in terms of incidence. A differentiated topography depending on the histological subtype of BCC was observed. The nodular histological subtype of BCC dominated in both cohorts, with the superficial multicentric subtype being the next most predominant on the scalp, followed by the infiltrative subtype on the ear. The head and neck regions were the most affected by BCC, especially in rural patients. In the future, the association between anatomical sites and the histological subtype may serve as a guide for personalised excision. The nasal and auricular regions were more susceptible to aggressive forms of BCC. Older patients develop larger lesions due to decreased DNA repair capacity. The mixed subtype of BCC was more common in the general cohort, with more aggressive histological subtypes. The Pinkus (fibroepithelial) histological subtype of BCC was identified exclusively in the thoracic region, thus making it a possible topographical indicator in certain cases. Tumour volume is directly proportional to patient age and indirectly to the year of diagnosis. The average tumour volume was smaller in the head and neck cohort. The volumes of the excision pieces were larger in men, and rural patients had larger tumour volumes in the head and neck cohort. Free excision margins are the norm in BCC diagnosis, thus confirming the effectiveness of BCC surgery. Recurrences were rare in both cohorts. The integration of all histological and topographical data may become the gold standard in personalising treatment for BCC, thereby supporting a model of personalised medicine even for cancers with low mortality rates.

## Figures and Tables

**Table 1:** Numerical and percentage distribution of simple and mixed BCC lesions in terms of histologic subtype and tumour characteristics (general cohort and head and neck cohort).

Variables		General Cohort			Head and Neck Cohort		
		Cases (n)	Cases (%)	*p*	Cases (n)	Cases (%)	*p*
Type of BCC							
Simple		84	15.55	<0.001	54	13.89	<0.001
Mixed		456	84.45		341	86.11	
Distribution of Simple and Mixed Types of BCC by Sex							
Mixed	Male	252	55.26	>0.05	188	55.13	>0.05
	Female	204	44.73		153	44.87	
Simple	Male	40	47.61		29	53.70	
	Female	44	52.38		25	46.30	

NA—not assessed, due to low-frequency categories.

**Table 2:** The distribution and statistical analysis of the histological subtype in relation to the simple or mixed type of BCC in the general cohort and head and neck cohort.

Variables		General Cohort				Head and Neck Cohort			
		Cases (n)		Cases (%)	*p*	Cases (n)		Cases (%)	*p*	
Type of BCC										
Simple		84		15.55	<0.001	54		13.89	<0.001	
Mixed		456		84.45		341		86.11		
Distribution of Simple and Mixed Types of BCC by Sex													
Mixed	Male	252		55.26		>0.05		188		55.13		>0.05	
	Female	204		44.73				153		44.87			
Simple	Male	40		47.61				29		53.70			
	Female	44		52.38				25		46.30			
**Variables**		**General Cohort**							**Head and Neck Cohort**				
		**Cases (n)**			**Cases (%)**		***p***	OR *	**Cases (n)**	**Cases (%)**	***p***		OR *
Histological Subtype													
Nodular		Simple	41		9.53		<0.001	6.53	36	10.97	<0.001		3.14
		Mixed	389		90.46				292	89.02			
Micronodular		Simple	1		0.88		<0.001	27.34	0	0	<0.001		∞ **
		Mixed	112		99.11				93	100			
Infiltrative		Simple	7		6.08		0.005	3.58	4	4.4	0.01		4.36
		Mixed	108		93.91				87	95.6			
Infundibulocystic		Simple	3		100		>0.05	-	3	100	0.02		0.02
		Mixed	0		0				0	0			
Adenoid Cystic		Simple	5		2.13		>0.05	-	3	1.6	<0.001		19.1
		Mixed	229		97.86				181	98.4			
Histological Characteristics													
Pillar Differentiation		Simple	4		3.92		<0.001	5.48	3	3.6	0.01		5.4
		Mixed	98		96.07				81	96.4			
Other associations between variables did not pass the statistical significance threshold *p* > 0.05													

* ORs are reported for mixed type of BCC; ** exclusive presence of micronodular subtype only in mixed BCC.

**Table 3:** Distribution and statistical analysis of histological subtype by patients’ gender and environment of origin in both study groups, with further subdivision within first general cohort.

General Cohort					Head and Neck Cohort				
Histological subtype	Gender *		*p* > 0.05		Histological subtype		Gender		*p* > 0.05
	Environment		*p* > 0.05				Environment		*p* > 0.05
Subtype			Other regions		Head and neck		Thorax		*p*
			n	%	n	%	n	%	
Superficial **		Absent	22	38.60	275	69.62	35	39.77	<0.001
		Present	35	61.40	120	30.38	53	60.23	
Pinkus *		Absent	57	100	395	100	86	97.73	0.058
		Present	0	0	0	0	2	2.27	

* Pinkus is only present in the general cohort at the thoracic level and only in female patients. ** Superficial even if it had a larger presence within the other regions. This is a cumulative subgroup, which ultimately means that the thoracic region is the most affected region.

**Table 4:** An analysis of the excision sites of the general cohort with the following variables: age, gender, environment of origin, years of diagnosis, histological subtype, histological characteristics, and macroscopic shapes of excisions and tumours.

Analysed Parameters		Head and Neck (n)	Thorax (n)	Other Regions (n)	*p*
Age–Median		*p* > 0.05			
Environment	Urban	266	82	36	0.0037
	Rural	120	15	21	
Gender		>0.05			
Years		>0.05			
Superficial subtype		117	56 *	35	<0.001
Elliptical Shape		278	77	14	0.0104
Irregular Shape		71	31	7	0.003

* the category “other regions” had the highest rate, but the number of cases in every distinct area is smaller so we considered the thoracic region to be predominantly present.

**Table 5:** A statistical analysis of the head and neck cohort in relation to histological subtypes, features, and excisional and macroscopical shapes.

Head and Neck Cohort	Variables		*p*-Value
	Histological subtype *	Excision sites	<0.001
	Histological features **	Excision sites	<0.001
	Elliptic excisional shape	Free resection margins	<0.001
	Elliptic excisional shape ***	Excision sites	<0.05
	Histological subtype ****	Macroscopic tumoural shape	<0.05
	Histological features	Macroscopic tumoural shape	>0.05

* The most frequent histological subtypes were as follows: nodular, adenoid cystic, and superficial. This applies to all excisional sites. ** The most frequent characteristics were as follows: free uninfiltrated margins; peritumoural inflammatory infiltrate; and ulcerations in only the nasal, ocular, auricular, and facial areas. *** An elliptical excisional shape was mainly found at the following sites: ocular, auricular, and facial. **** The nodular subtype mainly presented Elevated, Irregular, Round, Ulcerated, Nodular, and Linear shapes, while the superficial subtype was associated with a multinodular shape.

**Table 6:** The distribution of tumoural and excision volumes by size (<50 mm^3^ to >1000 mm^3^), gender (Male/Female), environment of origin (Rural/Urban), and year of diagnosis in the general cohort and head and neck cohort.

Volume mm^3^			General Cohort					Head and Neck Cohort				
			Piece			Tumour		Piece			Tumour	
			(n)	(%)		(n)	(%)	(n)	(%)		(n)	(%)
Very small	<50		28	5.2		246	47.58	26	6.6		194	51.73
Small	50–199		154	28.62		187	36.17	130	32.99		125	33.33
Medium	200–499		213	39.59		54	10.44	160	40.61		37	9.87
Big	500–999		90	16.73		16	3.09	52	13.2		8	2.13
Very big	>1000		53	9.85		14	2.71	26	6.6		11	2.93
Variables			General Cohort					Head and Neck Cohort				
			*p*		Volume—Median			*p*		Volume—Median		
Tumoural volume—Gender			0.4158		M: 56 mm^3^			0.6327		M: 48 mm^3^		
					F: 50.5 mm^3^					F: 48 mm^3^		
Tumoural volume—Environment			0.0764		R: 70 mm^3^			0.0126		R: 60 mm^3^		
					U: 50 mm^3^					U: 42 mm^3^		
Tumoural Volume—Year of diagnosis			<0.0001		2021: 99 mm^3^			<0.0001		2021: 89 mm^3^		
					2022: 56 mm^3^					2022: 49 mm^3^		
					2023: 75 mm^3^					2023: 54 mm^3^		
					2024: 30 mm^3^					2024: 30 mm^3^		
Excision volume—Gender			0.0189		M: 300 mm^3^			0.0077		M: 270 mm^3^		
					F: 264 mm^3^					F: 220 mm^3^		
Excision volume—Environment			0.8875		R: 276 mm^3^			0.1907		R: 264 mm^3^		
					U: 286 mm^3^					U: 234 mm^3^		
Excision volume—Year of diagnosis			0.0626		2021: 300 mm^3^			0.3008		2021: 270 mm^3^		
					2022: 302 mm^3^					2022: 220.5 mm^3^		
					2023: 300 mm^3^					2023: 250 mm^3^		
					2024: 253 mm^3^					2024: 234 mm^3^		
Parameters			General Cohort					Head and Neck Cohort				
			*p*		r			*p*		r		
Tumour volume		Age	<0.001		+0.149			0.0001		+0.195		
Tumour volume		Year diagnosis	<0.0001		−0.318			<0.0001		−0.311		
Excision volume		Age	0.0063		+0.118			0.0016		+0.159		
Excision volume		Year diagnosis	0.0110		−0.110			0.0876		−0.086		

**Table 7:** Volumetric analysis of cohorts according to BCC type and excisional areas.

Volume		Type—BCC	n	Median (mm^3^)	*p*-Value
General Cohort	Tumour	Mixed	439	56	*p* = 0.0889
		Simple	78	45	
	Excision	Mixed	455	286	*p* = 0.5173
		Simple	83	240	
Head and Neck Cohort	Tumour	Mixed	326	49	*p* = 0.1417
		Simple	49	39	
	Piece	Mixed	340	255	*p* = 0.1990
		Simple	54	204	
Volume		Site	n	Median (mm^3^)	*p*-value
General Cohort	Tumour	Head and neck	374	48	*p = 0*.0043
		Thoracic	96	93	
		Other sites	55	56	
	Piece	Head and neck	392	251	*p* < 0.0001
		Thoracic	96	485.5	
		Other sites	57	360	
Head and Neck Cohort	Tumour	** Auricular	18	39	*p* = 0.0681
		* Labial	8	121	
	Piece	* Labial	8	388	*p* < 0.0001
		** Nasal	127	182	

* the highest median; ** the lowest median.

**Table 8:** Head and neck cohort excision sites in relation to age.

Head and Neck Cohort						
Excision Sites	n	Minim	Q1	Median (Age)	Q3	Maxim
Facial **	121	27	60	67	75.5	90
Labial *	8	62	66	79.5	79.5	83
*p*	0.0172					

* highest median age; ** lowest median age.

**Table 9:** Analysis of tumoural and excisional volumes in relation to histological subtypes and histological features of BCC of general cohort and head and neck cohort.

Head and Neck Cohort							
Tumoural Volume	Histological subtype	Cases (n)	Mean (mm^3^)	Median (mm^3^)	SD (mm^3^)	Min (mm^3^)	Max (mm^3^)
	** Pigmented	12	94	37.5	170.8	150	625
	* Trichilemmal differentiation	2	201	201	72.12	3	252
	*p*—0.0573						
Analysis: Excision Volume—Histological Subtypes: *p* > 0.05							
Analysis: Tumoural Volume—Histological Characteristics: *p* > 0.05							
Analysis: Excision Volume—Histological Characteristics: *p* > 0.05							
General Cohort							
Analysis: Tumoural Volume—Histological Subtypes: *p* > 0.05							
Analysis: Excision Volume—Histological Subtypes: *p* > 0.05							
Analysis: Tumoural Volume—Histological Characteristics: *p* > 0.05							
Analysis: Excision Volume—Histological Characteristics: *p* > 0.05							

* the highest median. ** the lowest median.

## Data Availability

The data presented in this study are available on request from the first author and corresponding author due to ethical reasons.

## Supplementary Materials

The following supporting information can be downloaded at: https://www.mdpi.com/article/10.3390/diagnostics15182377/s1, Table S1. Numerical and percentage distribution of years of diagnosis and epidemiological data of patients with BCC included in the study (general cohort and head and neck cohort), Table S2. Numerical and percentage distribution of simple BCC lesions in terms of histologic subtype and tumour characteristics (general cohort and head and neck cohort), Table S3. Numerical and percentage distribution of mixed BCC lesions in terms of histologic subtype and tumour characteristics (general cohort and head and neck cohort), Table S4. Distribution and statistical analysis of the histological subtype in relation to the simple or mixed type of BCC in the general cohort and head and neck cohort, Table S5. Distribution and statistical analysis of histological subtype by patients’ sex in both the general cohort and the head and neck cohort, Table S6. Analysis of the excision sites of the general cohort with the following variables: age, gender, environment of origin, years of diagnosis, histological subtype, histological characteristics, macroscopic shapes of excisions and tumours, Table S7. Statistical analysis of the excision areas in the general cohort in relation to histological subtypes, Table S8. Statistical analysis of the excision areas in the head and neck cohort in relation to histological subtypes, Table S9. Statistical analysis of excision sites in relation to histological parameters in the head and neck cohort, Table S10. Statistical analysis of the excisional shapes in relation to the excision site and histological characteristics of the tumours within the head and neck cohort, Table S11. Statistical analysis of macroscopic tumoral shape in relation to histological subtypes within the head and neck cohort, Table S12. Distribution of tumoral volumes and excision pieces by gender (Male/Female), environment of origin (Rural/Urban), and year of diagnosis in the general cohort and head and neck cohort, Table S13. Statistical analysis of tumoral and excision volumes in relation to age and year of diagnosis in the general and head and neck cohort, Table S14. Analysis of tumor volume and excision pieces volume according to the type of BCC, simple or mixed, in both the general cohort and the head and neck cohort, Table S15. Analysis of tumoral volumes and pieces in relation to excision sites from the general cohort, Table S16. Analysis of tumoral volumes and excision piece in relation to histological subtypes and histological features of BCC of the general cohort and the head and neck cohort, Table S17. Head and neck cohort excision sites based on age.

## Abbreviations

The following abbreviations are used in this manuscript:

8-oxo-dG	8-oxo-7,8-dihydro-2-deoxyguanosine
BCC	Basal Cell Carcinoma
CHHP	Canonical Hedgehog Pathway
CI	Confidence Interval
CPD	Cyclobutene Pyrimidine Dimer
DHH	Desert Hedgehog
DNA	Deoxyribonucleic Acid
Gli	Glioma-associated Oncogene Homologue
HH	Hedgehog
IBM	International Business Machines
IHH	Indian Hedgehog
NA	Not Assessed
NMSC	Non-Melanocytic Skin Cancer
OR	Odds Ratio
P-HH	Hedgehog Protein
PTCH1	Patched Receptor 1
ROS	Reactive Oxygen Species
Rp-PTCH-1	Receptor Patch 1
Rp-SMO	Receptor Smoothened
SD	Standard Deviation
SHH	Sonic Hedgehog
SMO	Smoothened Receptor
SPSS	Statistical Package for the Social Sciences
UV	Ultraviolet
WHO	World Health Organization
nCHHP	Non-Canonical Hedgehog Pathway
