# Supplementary material for: The Epidemiological and Histopathological Profiling of Basal Cell Carcinoma: Insights from a 4-Year Institutional Cohort in a Romanian Clinical County Hospital
Source: Diagnostics (Basel). 2025 Sep 18;15(18):2377. doi: 10.3390/diagnostics15182377 (PMC12469015; doi:10.3390/diagnostics15182377)
Supplement: Supplementary file 1 [file diagnostics-15-02377-s001.zip › diagnostics-3832177-supplementary.pdf]

Supplementary material

Table S1 - Numerical and percentage distribution of years of diagnosis and epidemiological data of patients with BCC included in the study (general cohort and head and neck cohort)

Variables	GENERAL COHORT			HEAD AND NECK COHORT		
	Cases (n)	Cases (%)	p	Cases (n)	Cases (%)	p
YEAR OF DIAGNOSIS						
2021	103	19,07%	0,0031*	78	19,74%	<0,001*
2022	116	21,48%		86	21,77%	
2023	135	25,00%		97	24,55%	
2024	186	34,44%		134	33,92%	
ENVIRONMENT						
Urban	384	71,11%	<0,001*	273	69,11%	<0,001*
Rural	156	28,89%		122	30,89%	
SEX						
Male	292	54,07%	0,2008*	217	54,94%	0,0564*
Female	248	45,93%		178	45,06%	
AGE GROUPS (YEARS)						
20-29 years	1	0,19%	NA	1	0,25%	NA
30-39 years	7	1,30%		4	1,01%	
40-49 years	17	3,15%		17	4,29%	
50-59 years	40	7,41%		37	9,34%	
60-69 years	131	24,26%		125	31,57%	
70-79 years	135	25,00%		144	36,36%	
80-89 years	50	9,26%		64	16,16%	
90-100 years	3	0,56%		4	1,01%	

Table S2 - Numerical and percentage distribution of simple BCC lesions in terms of histologic subtype and tumour characteristics (general cohort and head and neck cohort)

Variables		GENERAL COHORT			HEAD AND NECK COHORT		
		Cases (n)	Cases (%)	p	Cases (n)	Cases (%)	p
TYPE OF BCC							
Simple		84	15,55%	<0,001	54	13,67%	<0,001
Mixed		456	84,45%		341	86,33%	
DISTRIBUTION OF SIMPLE AND MIXED TYPE OF BCC BY SEX							
Mixed	Male	252	55,26%	>0,05	188	55,13%	>0,05
	Female	204	44,74%		153	44,87%	
Simple	Male	40	47,62%		29	53,70%	
	Female	44	52,38%		25	46,30%	
SIMPLE BCC - DISTRIBUTION OF HISTOLOGICAL SUBTYPES, AND TUMOUR CHARACTERISTICS							
Nodular		41	48,81	NA	35	64,80%	NA
Micronodular		1	1,19		0	0%	

Adenoid- Cystic	5	5,95%		3	5,56%	
Infundibulocystic	3	3,57%		3	5,56%	
Infiltrative	7	8,33%		4	7,40%	
Superficial	25	29,76%		8	14,81%	
Keratotic	0	0%		0	0%	
Pigmented	0	0%		0	0%	
Trichilemmal differentiation	1	1,19%		1	1,85%	
Pinkus	1	1,19%		0	0%	
Squamous differentiation	0	0%	NA	0	0%	NA
Sebaceous differentiation	0	0%		0	0%	
Pillar Differentiation	4	4,76%		3	5,56%	
Recurrence	2	2,38%		2	3,70%	
Free margins	60	71,43%		39	72,22%	
Ulceration	15	17,86%		9	16,67%	
Intratumoral inflammatory infiltrate	0	0%		0	0%	
Peritumoral inflammatory infiltrate	38	45,24%		26	48,15%	
Perivascular invasion	0	0%		0	0%	
Perineural invasion	0	0%		0	0%	

NA – not assessed, due to low frequency categories

Table S3 - Numerical and percentage distribution of mixed BCC lesions in terms of histologic subtype and tumour characteristics (general cohort and head and neck cohort)

Variables		GENERAL COHORT			HEAD AND NECK COHORT		
		Cases (n)	Cases (%)	p	Cases (n)	Cases (%)	p
TYPE OF BCC							
Simple		84	15,55%	<0,001	54	13,89%	<0,001
Mixed		456	84,45%		341	86,11%	
DISTRIBUTION OF SIMPLE AND MIXED TYPE OF BCC BY SEX							
Mixed	Male	252	55,26%	0,2334	188	55,13%	>0,05
	Female	204	44,73%		153	44,87%	
Simple	Male	40	47,61%		29	53,70%	
	Female	44	52,38%		25	46,30%	
MIXED BCC - DISTRIBUTION OF HISTOLOGICAL SUBTYPES, AND TUMOUR CHARACTERISTICS							
Nodular		393	86,18%	NA	292	85,63%	NA
Micronodular		113	24,78%		93	27,27%	
Adenoid- Cystic		229	50,22%		181	53,08%	

Infundibulocystic	0	0%		0	0%	
Infiltrative	112	24,56%		87	25,51%	
Superficial	183	40,13%		112	32,84%	
Keratotic	31	6,80%		24	7,04%	
Pigmented	16	3,51%		13	3,81%	
Trichilemmal differentiation	1	0,22%		1	0,29%	
Pinkus	1	0,22%		0	0%	
Squamous differentiation	2	0,44%	NA	2	0,59%	NA
Sebaceous differentiation	1	0,22%		1	0,29%	
Pillar Differentiation	98	21,49%		81	23,75%	
Recurrence	4	0,88%		4	1,17%	
Free margins	365	80,04%		273	80,06%	
Ulceration	143	31,36%		110	32,26%	
Intratumorally inflammatory infiltrate	6	1,32%		5	1,47%	
Peritumoral inflammatory infiltrate	246	53,95%		184	53,96%	
Perivascular invasion	2	0,44%		2	0,59%	
Perineural invasion	0	0%		0	0%	

NA – not assessed, due to low frequency categories

Table S4 - Distribution and statistical analysis of the histological subtype in relation to the simple or mixed type of BCC in the general cohort and head and neck cohort.

Variables		GENERAL COHORT				HEAD AND NECK COHORT			
		Cases (n)	Cases (%)	p	OR*	Cases (n)	Cases (%)	p	OR*
HISTOLOGICAL SUBTYPE									
Nodular	Simple	41	9,53	<0,001	6,53	36	10,97	<0,001	3,14
	Mixed	389	90,46			292	89,02		
Micronodular	Simple	1	0,88	<0,001	27,34	0	0	<0,001	∞**
	Mixed	112	99,11			93	100		
Infiltrative	Simple	7	6,08	0,005	3,58	4	4,4	0,01	4,36
	Mixed	108	93,91			87	95,6		
Infundibulocystic	Simple	3	100	>0,05	-	3	100	0,02	0,02
	Mixed	0	0			0	0		
Adenoid-cystic	Simple	5	2,13	>0,05	-	3	1,6	<0,001	19,1
	Mixed	229	97,86			181	98,4		
HISTOLOGICAL CHARACTERISTICS									

Pillar Differentiation	Simple	4	3,92	<0,001	5,48	3	3,6	0,01	5,4
	Mixed	98	96,07			81	96,4		
Other associations between variables did not pass the statistical significance threshold p>0.05									

*OR are reported to mixed type of BCC

**due to the exclusive presence of Micronodular subtype only under mixed BCC

Table S5 - Distribution and statistical analysis of histological subtype by patients' sex in both the general cohort and the head and neck cohort

Subtype		GENERAL COHORT			HEAD AND NECK COHORT		
		Cases (n)	Cases (%)	p	Cases (n)	Cases (%)	p
GENDER							
Nodular	Female	197	45,81%	>0,05	153	46,64%	>0,05
	Male	233	54,19%		175	53,36%	
Micronodular	Female	60	51,28%		47	50,53%	
	Male	57	48,72%		46	49,47%	
Infundibulocysitic	Female	1	33,33%		1	33,33%	
	Male	2	66,67%		2	66,67%	
Adenoid-cystic	Female	96	41,03%		76	40,76%	
	Male	138	58,97%		108	59,24%	
Superficial	Female	103	49,76%		56	50,90%	
	Male	104	50,24%		64	49,10%	
Infiltrative	Female	49	42,61%		41	45,05%	
	Male	66	57,39%		50	54,95%	
Keratotic	Female	15	48,39%		14	58,33%	
	Male	16	51,61%		10	41,67%	
Pigmented	Female	6	37,50%		5	38,46%	
	Male	10	62,50%		8	61,54%	
Trichilemmal differentiation	Female	1	50%		1	50%	
	Male	1	50%		1	50%	
Pinkus*	Female	2	100%		-	-	
	Male	0	0%		-	-	
ENVIRONMENT OF ORIGIN							
Nodular	Rural	126	29,30%	>0,05	103	31,40%	>0,05
	Urban	304	70,70%		225	68,60%	
Micronodular	Rural	32	28,31%		28	30,10%	
	Urban	81	71,69%		65	69,90%	
Infundibulocysitic	Rural	0	0%		0	0%	
	Urban	3	100%		3	100%	
Adenoid-cystic	Rural	75	32,05%		63	34,24%	
	Urban	159	67,95%		121	65,76%	
Superficial	Rural	55	27,23%		38	31,67%	
	Urban	152	72,77%		82	68,33%	
Infiltrative	Rural	33	28,69%		24	26,37%	

	Urban	82	71,31%		67	73,63%	
Keratotic	Rural	6	19,35%		4	16,67%	
	Urban	25	80,65%		20	83,33%	
Pigmented	Rural	3	27,27%		3	23,07%	
	Urban	8	72,73%		10	76,93%	
Trichilemmal differentiation	Rural	1	50%		1	50%	
	Urban	1	50%		1	50%	
Pinkus*	Rural	0	0%		-*	-*	-*
	Urban	2	100%		-*	-*	-*

*Pinkus is only present in the general cohort (at a thoracic level)

Table S6. Analysis of the excision sites of the general cohort with the following variables: age, gender, environment of origin, years of diagnosis, histological subtype, histological characteristics, macroscopic shapes of excisions and tumours

GENERAL COHORT					
Analysed parameters		HEAD AND NECK (n)	THORAX (n)	OTHER REGIONS (n)	p
Age – Median		73	73	70	>0,05
Environment	Urban	266	82	36	0,0037
	Rural	120	15	21	
Gender	Female	179	40	33	>0,05
	Male	217	57	29	
Years	2021	74	18	11	>0,05
	2022	85	18	13	
	2023	94	27	14	
	2024	133	34	19	
Superficial subtype		117	56**	35	<0,001*
Elliptical Shape		278	77	14	0,0104*
Irregular Shape		71	31	7	0,003*

*Bonferroni correction applied to the Chi^2 test

** the category "other regions" had the highest rate, but the number of cases in every distinct area is smaller so we considered the thoracic region to be predominantly present

Table S7. Statistical analysis of the excision areas in the general cohort in relation to histological subtypes.

GENERAL COHORT								
Subtype		Other regions		Head and neck		Thorax		P
		n	%	n	%	n	%	
Nodular	Absent	14	24,56%	68	17,22%	24	27,27	>0,05
	Present	43	75,44%	327	82,78%	64	72,72	
Micronodular	Absent	49	85,96%	302	76,46%	75	85,23%	>0,05
	Present	8	14,04%	93	23,54%	13	14,77%	
Infundibulocystic	Absent	57	100%	392	99,24%	88	100%	>0,05
	Present	0	0%	3	0,76%	0	0%	

Adenoid-Cystic	Absent	39	68,42%	211	53,42%	56	63,64%	>0,05
	Present	18	31,58%	184	46,58%	32	36,36%	
Superficial	Absent	22	38,60%	275	69,62	35	39,77	<0,001
	Present	35	61,40%	120	30,38	53	60,23	
Infiltrative	Absent	45	78,95%	304	76,96%	72	81,82%	>0,05
	Present	12	21,05%	91	23,04%	16	18,18%	
Keratotic	Absent	56	98,25%	371	93,92%	82	93,18%	>0,05
	Present	1	1,75%	24	6,08%	6	6,82%	
Pigmented	Absent	55	96,49%	382	96,71%	87	98,86%	>0,05
	Present	2	3,51%	13	3,29%	1	1,14%	
Trichilemmal differentiation	Absent	57	100%	393	99,49%	88	100%	>0,05
	Present	0	0%	2	0,051%	0	0%	
Pinkus	Absent	57	100%	395	100%	86	97,73	0,058
	Present	0	0%	0	0%	2	2,27%	

Table S8. Statistical analysis of the excision areas in the head and neck cohort in relation to histological subtypes

HEAD AND NECK COHORT							
Subtype	Excision site (n)						
	Nasal	Ocular	Scalp	Auricular	Facial	Labial	Cervical
Nodular	115	36	24	17	105	7	7
Micronodular	27	9	9	4	34	2	2
Infudibulocystic	0	0	2	0	1	0	0
Adenoid-Cystic	66	23	13	8	59	6	6
Superficial	34	8	20	11	37	0	0
Infiltrative	32	7	7	11	25	2	2
Keratotic	11	1	1	0	8	1	1
Pigmented	2	0	3	3	3	0	0
Trichilemmal differentiation	1	0	0	1	0	0	0
p	<0,001	<0,001	<0,001	<0,001	<0,001	<0,001	<0,001

*Fisher test was performed for the labial and cervical region. Chi^2 test (with Bonferroni correction applied) was performed for the remaining regions.

Table S9 - Statistical analysis of excision sites in relation to histological parameters in the head and neck cohort

HEAD AND NECK COHORT				
Characteristics	Excision sites (n)			
	Nasal	Ocular	Auricular	Facial
Recurrence	2	1	1	2
Pillar differentiation	31	5	8	26

Squamous differentiation	2	0	0	0
Ulceration	33	18	11	37
Sebaceous differentiation	1	0	0	0
Free uninfilitrate margins	105	29	16	103
Inflammatory infiltrate Intratumoral	3	0	0	0
Inflammatory infiltrate Peritumoral	63	22	14	69
Perineural invasion	0	0	0	0
Perivascular invasion	2	0	0	0
p	<0,001	<0,001	<0,001	<0,001

* Monte Carlo test was performed for the facial and auricular region. Chi^2 test (with Bonferroni correction applied) was performed for the remaining regions

Table S10. Statistical analysis of the excisional shapes in relation to the excision site and histological characteristics of the tumours within the head and neck cohort

HEAD AND NECK SUBROUP				
EXCISION SHAPE	EXCISION SITE (n)			HISTOLOGIC FEATURES (n)
	Ocular	Auricular	Facial	Free resection margins
Elliptic	22	10	106	238
Multiple fragments	1	1	1	1
Unspecified	10	3	9	23
Irregular	8	7	3	36
Oval	0	1	0	2
Round-oval	5	1	1	8
Round	1	0	3	5
p*	0,0217	0,0476	<0,001	<0,001

* Fisher's test (with Bonferroni correction applied) was performed for all regions. For the remaining features and excision areas, there were no statistically significant differences (p>0.05)

Table S11. Statistical analysis of macroscopic tumoral shape in relation to histological subtypes within the head and neck cohort

HEAD AND NECK COHORT								
Subtype	Macroscopic tumoral shape (n)							
	Elevated (n)	Irregular (n)	Round (n)	Ulcerated (n)	Nodular (n)	Unspecified (n)	Multinodular (n)	Linear (n)
Nodular	60	56	19	24	102	33	4	6
Micronodular	12	16	6	6	36	5	0	2
Infudibulocystic	0	0	2	0	0	0	1	0
Adenoid-Cystic	37	26	8	15	74	11	3	0
Superficial	18	27	7	8	27	15	6	1
Infiltrative	13	21	6	10	20	8	1	3
Keratotic	3	7	0	1	8	2	1	1
Pigmented	3	3	0	0	2	3	1	0

Trichilemmal differentiation	0	0	0	1	0	1	0	0
p	<0,001	<0,001	<0,001	<0,001	<0,001	<0,001	0,039	0,003

* Histological features showed no significant differences in tumoral shape (p>0.05)

Table S12. Distribution of tumoral volumes and excision pieces by gender (Male/Female), environment of origin (Rural/Urban), and year of diagnosis in the general cohort and head and neck cohort

**Volume mm^3**		**GENERAL COHORT**				**HEAD AND NECK COHORT**			
		Piece		Tumour		Piece		Tumour	
		(n)	(%)	(n)	(%)	(n)	(%)	(n)	(%)
**Very small**	<50	28	5,2	246	47,58	26	6,6	194	51,73
**Small**	50-199	154	28,62	187	36,17	130	32,99	125	33,33
**Medium**	200-499	213	39,59	54	10,44	160	40,61	37	9,87
**Big**	500-999	90	16,73	16	3,09	52	13,2	8	2,13
**Very big**	>1000	53	9,85	14	2,71	26	6,6	11	2,93

Volume (mm^3)			Cases (n)	Mean (mm^3)	Median (mm^3)	SD (mm^3)	Min (mm^3)	Max (mm^3)
GENERAL COHORT	Tumor	M	281	156,2	56	345,3	3	3381
		F	236	157	50,5	420,9	1	3933
		p = 0,4158						
	Piece	M	291	770,8	300	3666	6	60750
		F	247	630,9	264	3581	6	56000
		p = 0,0189						
	Tumor	R	147	179,3	70	393,1	1	3672
		U	370	147,6	50	376,7	2	3933
		p = 0,0764						
	Piece	R	155	877	276	4539	6	56000
		U	383	637,6	286	3185	6	60750
		p = 0,8875						
	Tumor	2021	96	256,2	99	584,5	3	3933
		2022	109	126,3	56	167,3	2	868
		2023	131	220	75	469	2	3672
		2024	181	76,15	30	213,5	1	2640
		p <0,0001*						
	Piece	2021	103	1107	300	5548	6	56000
		2022	116	435,8	302	462,4	45	2800
		2023	134	1060	300	5328	6	60750
		2024	185	390,7	253	545,3	12	4620
		p=0,0626*						

Volume (mm^3)			Cases (n)	Mean (mm^3)	Median (mm^3)	SD (mm^3)	Min (mm^3)	Max (mm^3)
HEAD AND NECK	Tumor	M	208	128,1	48	274,4	3	2640
		F	167	174,2	48	492,6	2	3933
		p – 0,6327						
	Piece	M	217	444,7	270	696,2	6	6048

		F	177	370,5	220	622	6	4785
		p – 0,0077						
	Tumor	R	115	183,8	60	417,1	3	3672
		U	260	133,1	42	372,9	2	3933
		p – 0,0126						
	Piece	R	121	518,5	264	943,1	6	6048
		U	273	363,9	234	487,4	6	4785
		p – 0,1907						
	Tumor	2021	72	240,8	89	554,3	3	3933
		2022	79	114,4	49	167,7	4	868
		2023	95	202,8	54	492,6	2	3672
		2024	129	78,37	30	244,1	3	2640
		p < 0,0001*						
	Piece	2021	78	416,6	270	448,7	6	2750
		2022	86	362,8	220,5	345,2	45	1575
		2023	97	529,4	250	1040	6	6048
		2024	133	353,6	234	563	12	4620
		p – 0,3008*						

Test Mann-Whitney, *Test Kruskal-Wallis

Table S13. Statistical analysis of tumoral and excision volumes in relation to age and year of diagnosis in the general and head and neck cohort

Parameters		GENERAL COHORT		HEAD AND NECK COHORT	
		p	r	p	r
Tumor volume	Age	<0,001	+0,149	0,0001	+0,195
Tumor volume	Year diagnosis	<0,0001	-0,318	<0,0001	0,311
Tissue sample volume	Age	0,0063	+0,118	0,0016	+0,159
Tissue sample volume	Year diagnosis	0,0110	-0,110	0,0876	-0,086

Table S14. Analysis of tumor volume and excision pieces volume according to the type of BCC, simple or mixed, in both the general cohort and the head and neck cohort.

Volume		Type	n	Mean (mm^3)	Median (mm^3)	SD (mm^3)	Min (mm^3)	Max (mm^3)
GENERAL	Tumor	Mixed	439	169,3	56	410,1	1	3932
		Simple	78	85,32	45	108,6	2	537
	p = 0,0889							
	Piece	Mixed	455	739,6	286	3915	6	60750
		Simple	83	525,2	240	1061	15	9120
	p = 0,5173							
HEAD	Volume	Type	n	Mean (mm^3)	Median (mm^3)	SD (mm^3)	Min (mm^3)	Max (mm^3)

	Tumor	Mixed	326	158,2	49	412	2	3933
		Simple	49	85,18	39	119	3	539
	p = 0,1417							
	Piece	Mixed	340	420,1	255	689	6	6048
		Simple	54	356,3	204	479,9	15	2912
	p = 0,1990							

Table S15. Analysis of tumoral volumes and pieces in relation to excision sites from the general cohort

Volume		Site	n	Mean (mm^3)	Median (mm^3)	SD (mm^3)	Min (mm^3)	Max (mm^3)
GENERAL COHORT	Tumor	Head and neck	374	148,7	48	387,7	2	3931
		Thoracic	96	184	93	375,2	1	3380
		Other sites	55	156,3	56	321,2	2	1748
	p – 0,0043							
	Piece	Head and neck	392	410,3	251	665,5	6	6048
		Thoracic	96	768,4	485,5	1312	77	9240
		Other sites	57	2595	360	10758	40	60750
	p < 0,0001							

Table S16. Analysis of tumoral volumes and excision piece in relation to histological subtypes and histological features of BCC of the general cohort and the head and neck cohort

HEAD AND NECK COHORT								
Tumoral Volume	Histological subtype	Cases (n)	Mean (mm^3)	Median (mm^3)	SD (mm^3)	Min (mm^3)	Max (mm^3)
	Nodular	311	147,1	42	405,6	2	3933
	Micronodular	88	153,3	50,5	436,1	3	3933
	Infudibulocystic	3	101,3	88	72,92	36	180
	Adenoid-cystic	179	206,2	56	514,9	6	3933
	Superficial	115	115,3	45	251,7	3	1728
	Infiltrative	87	126,8	54	272,3	4	2125
	Keratotic	23	70,28	50	73,55	9	270
	Pigmented	12	94	37,5	170,8	150	625
	Trichilemmal differentiation	2	201	201	72,12	3	252
	p – 0,0573						
Analysis: Piece Volume – Histological Subtypes = p > 0.05							
Analysis: Tumor Volume – Histological Characteristics = p > 0.05							
Analysis: Piece Volume – Histological Characteristics = p > 0.05							
GENERAL COHORT							

	Histological subtype	Cases (n)	Mean (mm^3)	Median (mm^3)	SD (mm^3)	Min (mm^3)	Max (mm^3)
Tumoral Volume	Nodular	416	158,8	50	407,7	1	3933
	Micronodular	109	144,9	51	397,5	1	3933
	Infudibulocystic	3	101,3	88	72,92	36	180
	Adenoid-cystic	228	210,3	62	515,4	2	3933
	Superficial	201	129,6	54	243,7	1	1750
	Infiltrative	114	142,8	60	263,1	3	2125
	Keratotic	30	96,08	43	132,2	4	608
	Pigmented	15	90,6	35	156,5	9	625
	Trichilemmal differentiation	2	201	201	72,12	150	252
	Pinkus	2	150	150	127,3	60	240
	p – 0,1328						
Analysis: Tumor Volume – Histological Subtypes = p > 0.05							
Analysis: Tumor Volume – Histological Characteristics = p > 0.05							
Analysis: Tumor Volume – Histological Subtypes = p > 0.05							

Table S17. Head and neck cohort excision sites based on age

HEAD AND NECK COHORT						
Excision sites	n	Minim	Q1	Median (age)	Q3	Maxim
Nasal	127	45	65	72	77	88
Ocular	45	37	63,5	70	79,5	91
Scalp	30	44	62,75	71	80,5	85
Auricular	20	61	69,25	75	81,5	93
Facial**	121	27	60	67	75,5	90
Labial*	8	62	66	79,5	79,5	83
Cervical	27	47	65	78	78	85
p	0,0172					

*Highest median age

**lowest median age
